# Supplementary material for: Thiacalixarene Carboxylic Acid Derivatives as Inhibitors of Lysozyme Fibrillation
Source: Int J Mol Sci. 2024 Apr 26;25(9):4721. doi: 10.3390/ijms25094721 (PMC11083589; doi:10.3390/ijms25094721)
Supplement: Supplementary file 1 [file ijms-25-04721-s001.zip › ijms-2962459-supplementary.pdf]

Thiacalixarene Carboxylic Acid Derivatives as Inhibitors of Lysozyme Fibrillation

Anastasia Nazarova[1], Igor Shiabiev[1], Ksenia Shibaeva[1], Olga Mostovaya[1], Timur Mukhametzyanov[1], Arthur Khannanov[1], Vladimir Evtugyn[2], Pavel Zelenikhin[3], Xiangyang Shi[4,5], Mingwu Shen[4], Pavel Padnya[1,*], and Ivan Stoikov[1,*]

[1] A. M. Butlerov Chemistry Institute, Kazan Federal University, 18 Kremlyovskaya str., 420008 Kazan, Russia;

[2] Interdisciplinary Center of Analytical Microscopy, Kazan Federal University, 18 Kremlyovskaya str., 420008 Kazan, Russia

[3] Institute of Fundamental Medicine and Biology, Kazan Federal University, 18 Kremlyovskaya str., 420008 Kazan, Russia;

[4] State Key Laboratory for Modification of Chemical Fibers and Polymer Materials, Shanghai Engineering Research Center of Nano-Biomaterials and Regenerative Medicine, College of Biological Science and Medical Engineering, Donghua University, Shanghai 201620, People's Republic of China;

[5] CQM - Centro de Química da Madeira, Universidade da Madeira, Campus Universitário da Penteada, 9020-105 Funchal, Portugal

* Correspondence: padnya.ksu@gmail.com (P.P.); ivan.stoikov@mail.ru (I.S.); Tel.: +7-843-233-7241 (I.S.)

Electronic Supplementary Information (28 pages)

Table of Contents

1.	Materials and Methods	3
2.	^{1}H NMR spectra	7
3.	$^{13}C\{^{1}H\}$ NMR spectra	8
4.	ESI mass-spectra	10
5.	FTIR spectra	11, 26–28
6.	DLS data	13–16, 23–25
7.	Cytotoxicity	17
8.	UV-Vis spectra	18
9.	Job’s plots	19
10.	Bindfit data	20
11.	Fluorescence spectra	25
12.	References	28

S1. Materials and Methods

S1.1. General

^{1}H and $^{13}C\{^{1}H\}$ NMR spectra were obtained on a Bruker Avance-400 spectrometer (Bruker Corp., Billerica, MA, USA) ($^{13}C\{^{1}H\}$ – 100 MHz and ^{1}H – 400 MHz). The chemical shifts were determined against the signals of residual protons of deuterated solvent (D_2O). The concentrations of the compounds were equal to 3–5% by the weight in all the records. The FTIR spectra were recorded on the Spectrum 400 FTIR ATR spectrometer (Perkin Elmer Inc, Waltham, MA, USA) with a Diamond KRS-5 attenuated total internal reflectance attachment (resolution 0.5 cm^{-1}, accumulation of 64 scans, recording time 16 s in the wavelength range 400–4000 cm^{-1}). ESI-HRMS experiments were performed at Agilent 6550 iFunnel Q-TOF LC/MS (Agilent Technologies, Santa Clara, CA, USA), equipped with Agilent 1290 Infinity II LC. Melting points were determined using Boetius Block apparatus (VEB Kombinat Nagema, Radebeul, Germany).

Chemically pure organic solvents were purified by standard methods. All the aqueous solutions were prepared with the Millipore-Q deionized water (>18.0 MΩ × cm at 298 K).

S1.2. Dynamic light scattering (DLS)

S1.2.1. Particles' size

The distribution of particles by number, volume, and intensity, the polydispersity index were determined by dynamic light scattering (DLS) on a Zetasizer Nano ZS instrument (Malvern Instruments, Worcestershire, UK) in quartz cuvettes. The instrument is equipped with the 4 mW He-Ne laser (633 nm). Measurements were performed at a detection angle of 173°. The error in determining the particle size is less than 2%. The results were processed by the DTS program (Dispersion Technology Software 4.20). Deionized water was used to prepare solutions for studying the aggregation of **TOPA** macrocycles. In the course of the experiment, the concentrations of macrocycles varied within 1 – 100 μM. Double systems (thiacalix[4]arene-HEWL) were prepared similarly to those studied by UV-Vis spectroscopy. The particle sizes were measured after 1 h mixing. Measurements were determined after 24 and 178 h three times to evaluate kinetic stability.

S1.3. Electrophoretic light scattering (ELS)

S1.3.1. Zeta potentials

Zeta (ζ) potentials were determined by electrophoretic light scattering (ELS) on a Zetasizer Nano ZS from Malvern Instruments (Worcestershire, UK). Samples

were prepared as for the DLS measurements and were transferred with the syringe to the disposable folded capillary cell for measurement. The zeta potentials were measured using the Malvern M3-PALS method and averaged from three measurements.

*S1.4. Cytotoxicity of **TOPA** on A549 cell line*

The ability of **TOPA** to inhibit the viability and proliferative activity of A549 cells was investigated using the MTT test according to [S1]. A549 – human alveolar adenocarcinoma cell line (ATCC, Rockville, Maryland, USA) was cultured in DMEM (GIBCO, Waltham, MA, USA) medium supplemented with 10% serum and 100 units/mL of penicillin (PanEco, Moscow, Russia) and streptomycin (PanEco, Moscow, Russia) in a humidified atmosphere with 5% CO_2 at 37 °C. Cells were seeded in 96-well plates at a concentration of 10^4 cells/well. After 24 h of cultivation, the medium was removed from the wells and replaced with a fresh one with the addition of the test substances. The volume of the culture medium in the wells was 100 µL. After 24 h incubation of cells in the presence of substances, the medium in the wells was replaced with a fresh one containing MTT reagent at a final concentration of 0.5 mg/mL. The cells were incubated with MTT for 4 h at 37 °C in an atmosphere of 5% CO_2. Then, the medium was aspirated from the wells and 100 µL of dimethyl sulfoxide was added. Probes were incubated at 37 °C for 15 min in the dark for the formazan crystals to dissolve. The optical density of the formazan solution in the wells was measured using a plate reader (BioRad xMarkTM Microplate Spectrophotometer, USA) at a wavelength of 570 nm. Three series of experiments were carried out with at least 8 replications for each variant in the series. For MTT test data significant differences were reported at $p < 0.05$ using the nonparametric Mann-Whitney U-test.

S1.5. UV-Vis spectroscopy

Absorption spectra were recorded on a Shimadzu UV-3600 spectrometer (Kyoto, Japan). Quartz cuvettes with an optical path length of 10 mm were used. Deionized water was used for preparation of the solutions. 300 µL solution of HEWL (10 µM) was added to water solution of **TOPA** (10 µM) in a 1:10 ratio to study the complexation of thiacalix[4]arenes with the guest in solvent. Absorption spectra of mixtures were recorded after an 1 h incubation at 20 °C.

S1.5.1. Determination of the stability constant and stoichiometry of the complex by spectrophotometric titration

The solution (10 μM) of HEWL (100, 200, 300, 400, 500, 600, 700, 800, and 900 μL) in water was added to 300 μL of a solution of **TOPA** (100 μM) in water and diluted to final volume of 3 mL with water. The UV-Vis spectra of the solutions were then recorded. The stability constant of complex was calculated by Bindfit application (Bindfit v0.5, Open Data Fit, http://app.supramolecular.org/bindfit/). Three independent experiments were carried out for each series.

S1.6. Circular dichroism spectroscopy

Circular dichroism (CD) spectra were recorded on a Jasco J-1500CD spectrophotometer (Tokyo, Japan) in a quartz cuvette with an optical path length of 10 mm. 20 mM phosphate buffer (PBS) (pH 7.44) containing 20 mM NaCl was used for preparation of the solutions. The experiment was performed at 25 °C, scanning wavelength range was 200–250 nm. The scanning rate was 20 nm/min. The concentrations of substances in the CD measurements for HEWL and **TOPA** were equal to 0.5 μM. The CD spectra of mixtures were recorded after an 1 h incubation at 25 °C.

S1.7. Transmission and scanning electron microscopy

TEM and SEM measurements were made at Interdisciplinary Center for Analytical Microscopy of Kazan Federal University. Analysis of samples was carried out using a Hitachi HT7700 Exalens atomic transmission electron microscope (Tokyo, Japan) with an Oxford Instruments X-Maxn 80T EDS detector working in STEM mode and a Merlin (Carl Zeiss) universal analytical scanning auto-emission electron microscopy system. Samples of double systems thiacalix[4]arene **TOPA**-HEWL were prepared similarly to those studied by the DLS method. Deionized water was used as a solvent. The suspension (10 μL) was placed on a carbon-coated 3 mm copper grid and dried at room temperature using special holder for microanalysis. After drying, the grid was placed in the transmission electron microscope and analyzed at an accelerating voltage of 100 kV.

S1.8. Preparation of fibrillar solutions of HEWL

HEWL powder (50 mg) was dissolved in 1 mL deionized water. The concentration of the stock HEWL solution was determined spectrophotometrically by HEWL absorbance at 280 nm using a molar extinction coefficient of 37646 $M^{-1}\times cm^{-1}$ [S2]. HEWL concentration found to vary in the range of 3.50 ± 0.06 mM (50.5 ± 0.8 mg/mL). HEWL solutions with a concentration of ~150 μM were

prepared by diluting the stock solution in 20 mM phosphate buffer (PBS) (pH 7.44) containing 20 mM NaCl and 30% ethanol with and without different concentrations (75, 150, and 300 μM) of **TOPA**. Incubation of solutions was carried out for 6 h at 65 °C. Then incubated protein solutions were left for 24 hours at room temperature prior to experimental measurements. Aliquots were diluted with 20 mM PBS in each study to maintain the desired concentration in the final working solution.

S1.9. Fluorescence spectroscopy

Fluorescence spectra were recorded on the Fluorolog 3 luminescent spectrometer (Horiba Jobin Yvon, Longjumeau, France). The excitation wavelength was selected as 240 nm. The emission scan range was 460–600 nm. Excitation and emission slits were 3 nm. Quartz cuvettes with an optical path length of 10 mm were used. The cuvette was placed at the front face position to avoid the inner filter effect. Fluorescence spectra were automatically corrected by the Fluorescence program. The fluorescence spectra of ThT (10 μM) in presence and absence of HEWL fibrillar solutions (5 μM) were recorded in 20 mM PBS (pH 7.44) at 293 K. Three independent experiments were carried out for each series.

S1.10. FTIR Spectroscopy

The FTIR spectra were recorded on a Spectrum 400 (PerkinElmer, Waltham, MA, USA) FTIR spectrometer, with Diamond KRS-5 ATR accessory. The FTIR spectra from 4000 to 400 cm^{-1} were considered in this analysis. The spectra were measured with 0.5 cm^{-1} resolution and 32-scan accumulation. Thiacalixarenes were used as a baseline in experiments assessing the inhibition of fibril formation to neutralize the effect of amide groups on the fibril α-helices. The aqueous solution was applied directly to the sensor and allowed to air dry naturally. After that, the spectra were recorded. The spectra were cleared of noise by the built-in Perkin Elmer software (Version 10), without baseline correction. To assess the effect of thiacalix[4]arenes on fibril formation, deconvolution of the IR spectra was carried out, with a constant baseline line for all studied spectra. The ratios of α-helices and β-sheets were calculated to evaluate the influence after deconvolution.

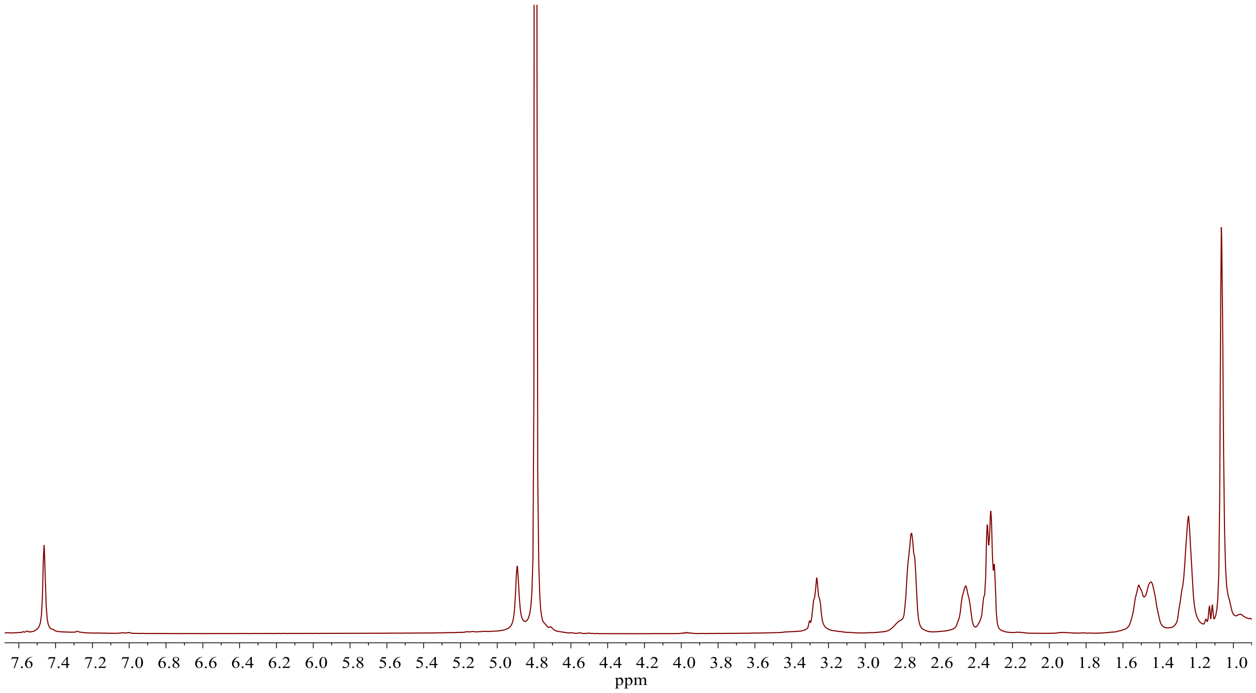

Fig. S1. ^{1}H NMR spectrum of TOPA-cone, D_2O, 298 K, 400 MHz.

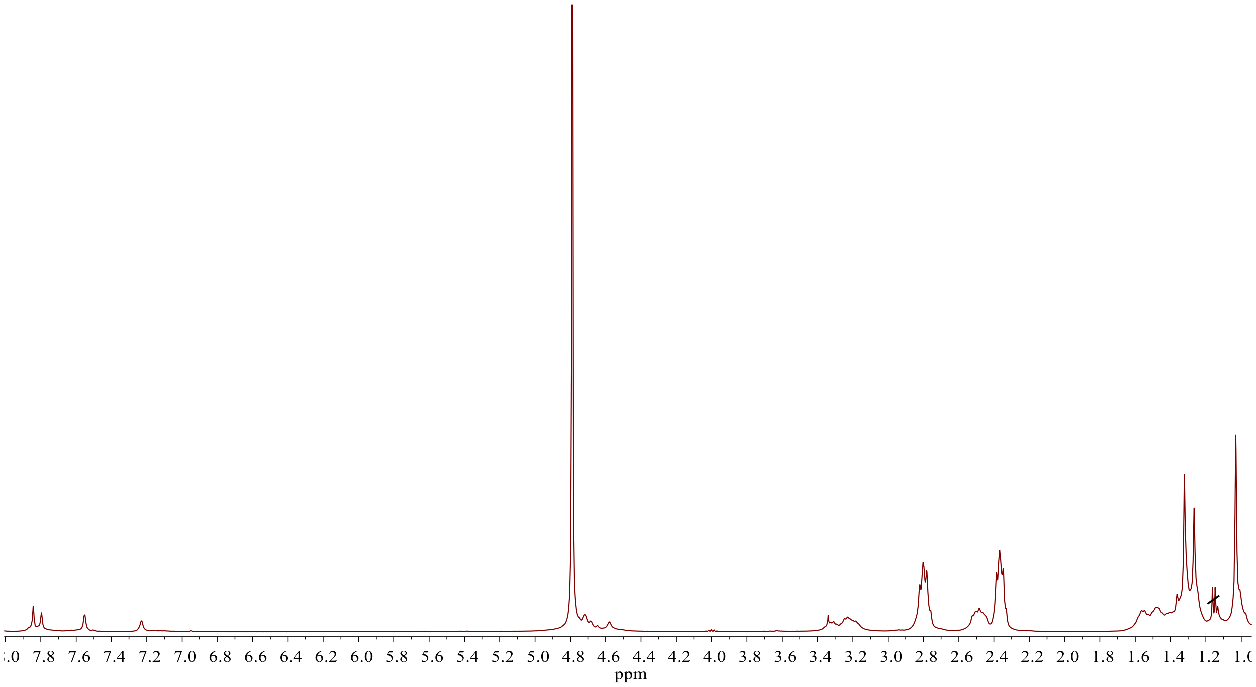

Fig. S2. ^{1}H NMR spectrum of TOPA-paco, D_2O, 298 K, 400 MHz.

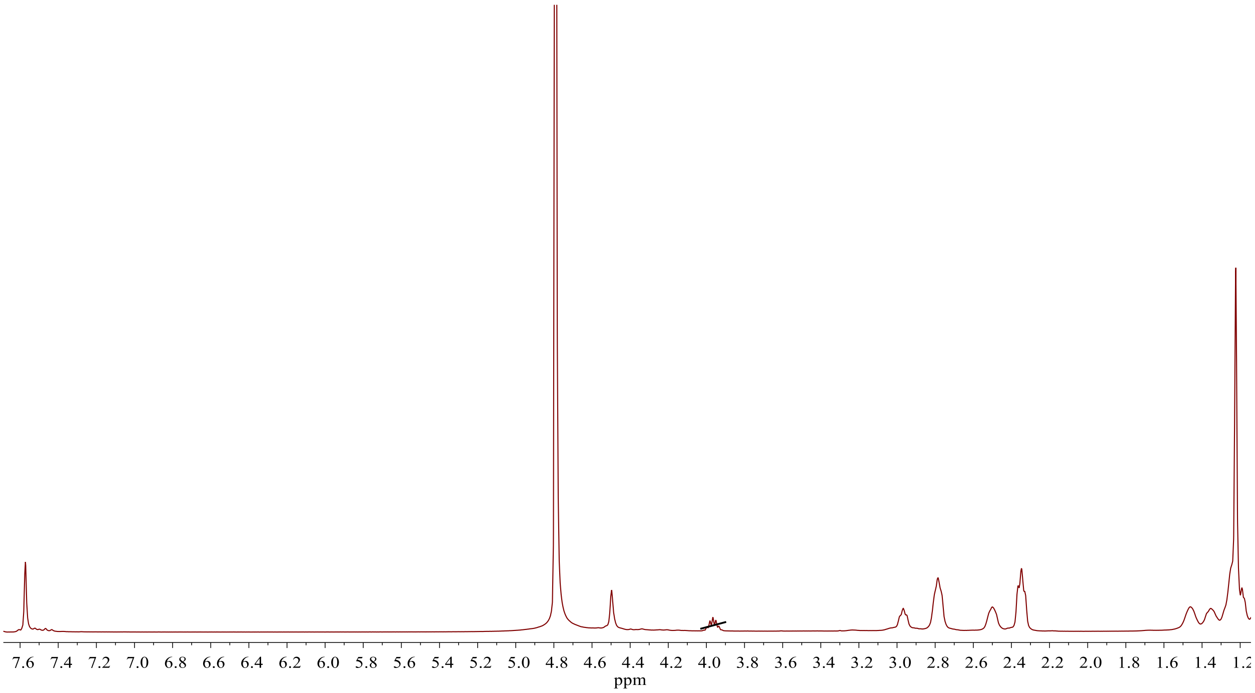

Fig. S3. **^{1}H NMR spectrum of TOPA-alt, D_2O, 298 K, 400 MHz.**

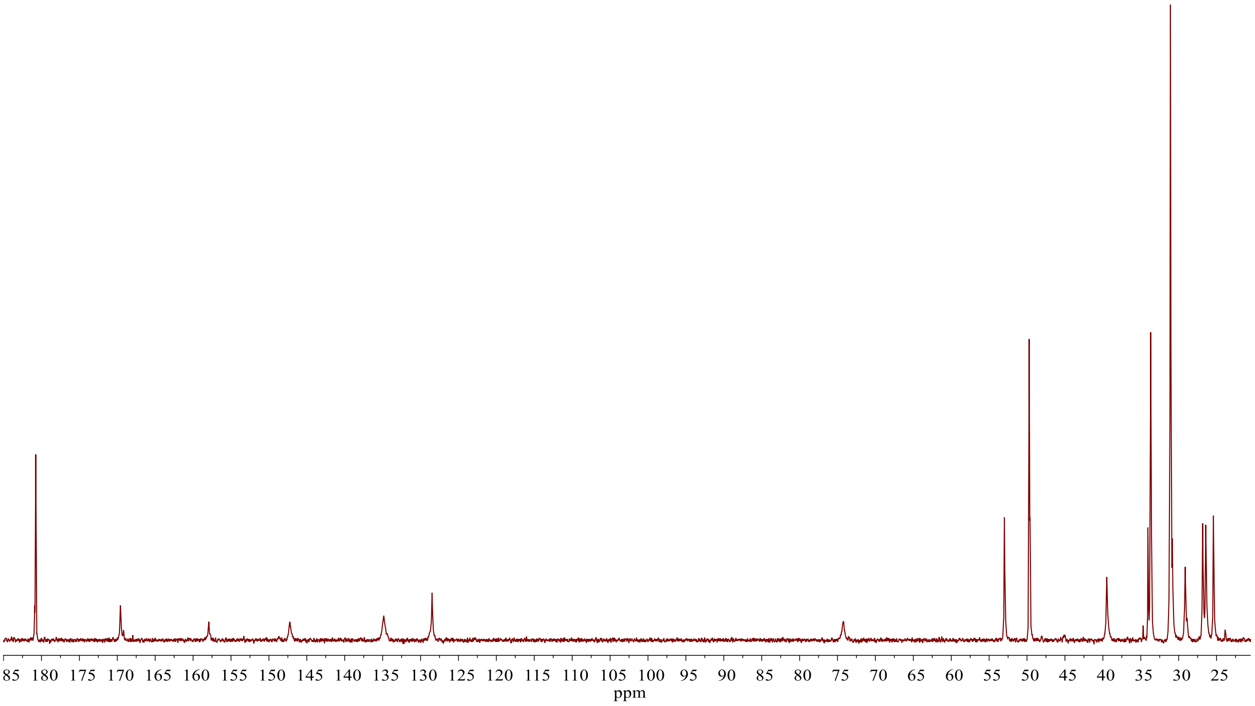

Fig. S4. **^{13}C{^{1}H} NMR spectrum of TOPA-cone, D_2O, 298 K, 100 MHz.**

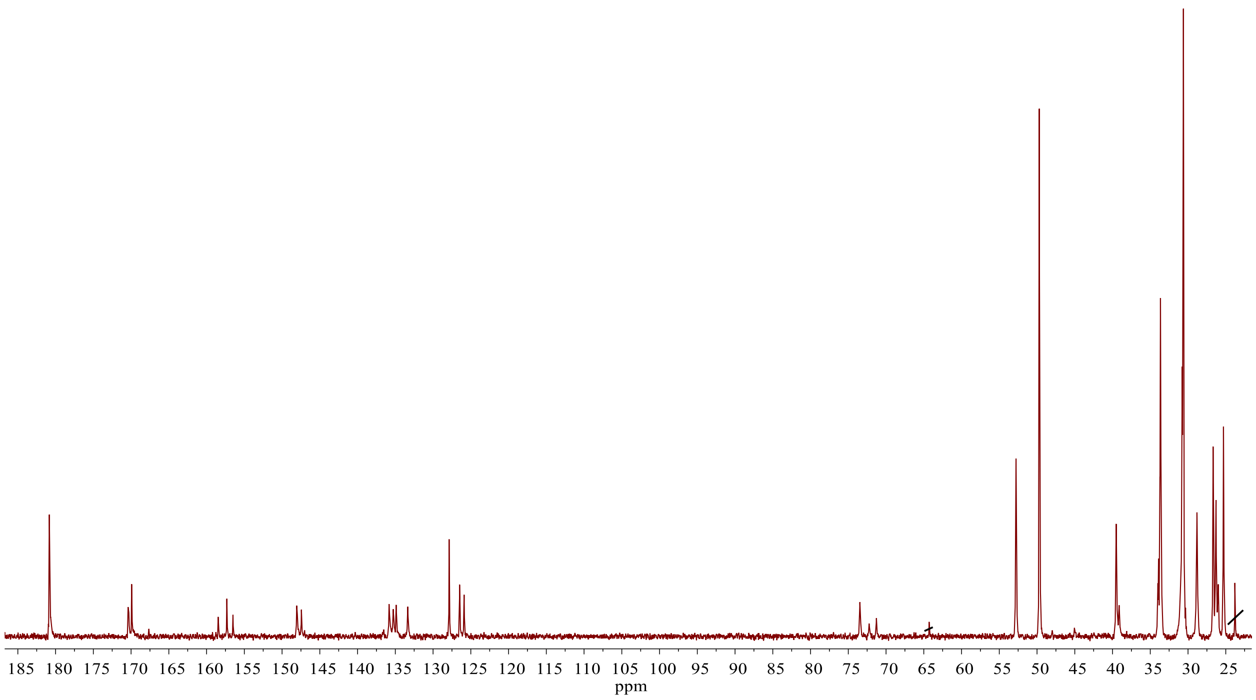

Fig. S5. $^{13}C\{^1H\}$ NMR spectrum of TOPA-paco, D_2O, 298 K, 100 MHz.

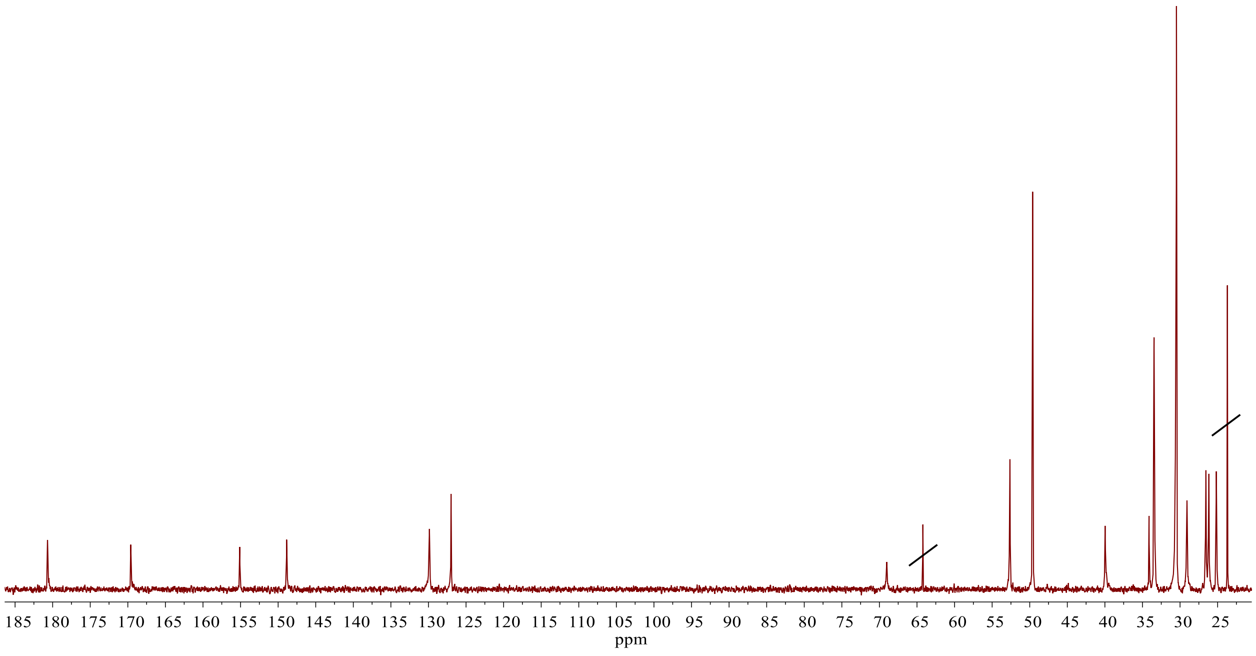

Fig. S6. $^{13}C\{^1H\}$ NMR spectrum of TOPA-alt, D_2O, 298 K, 100 MHz.

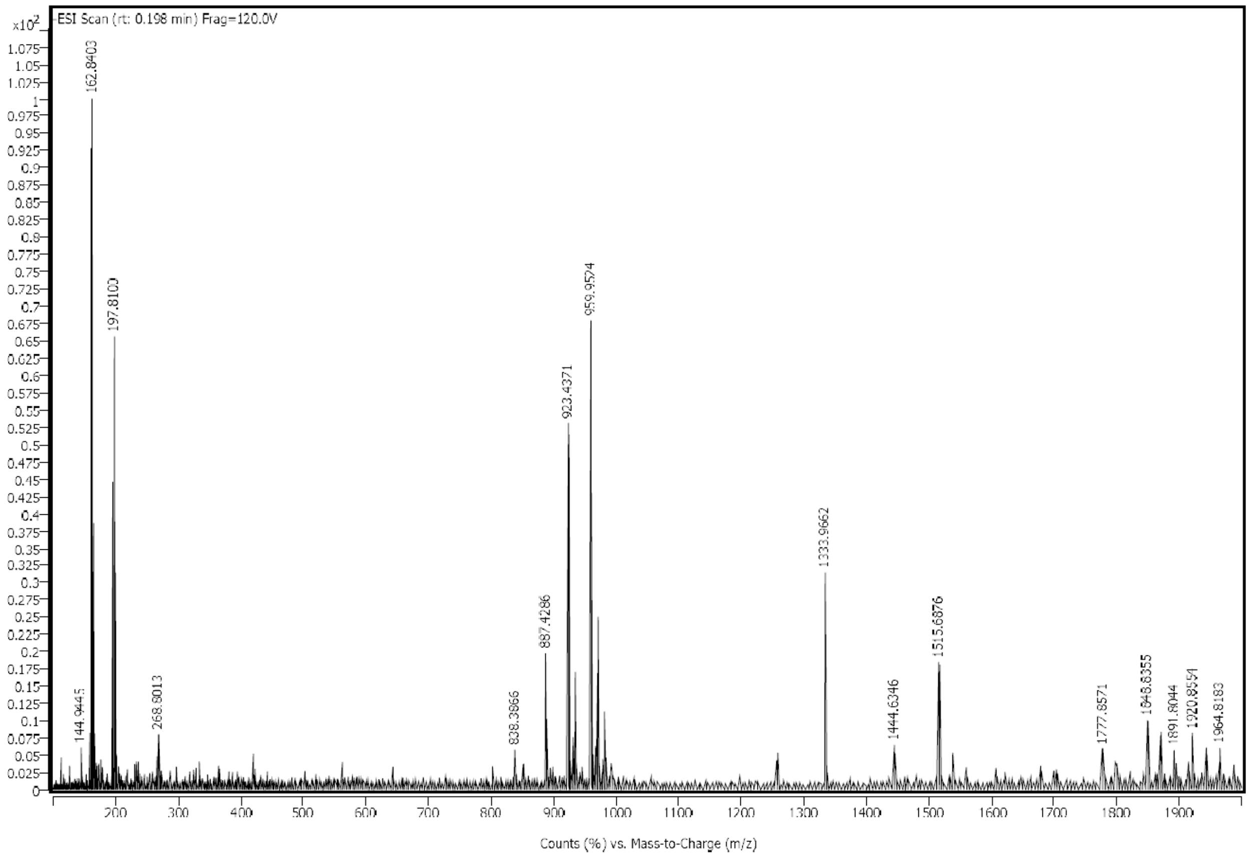

Fig. S7. Mass spectrum (HRMS ESI) of TOPA-cone.

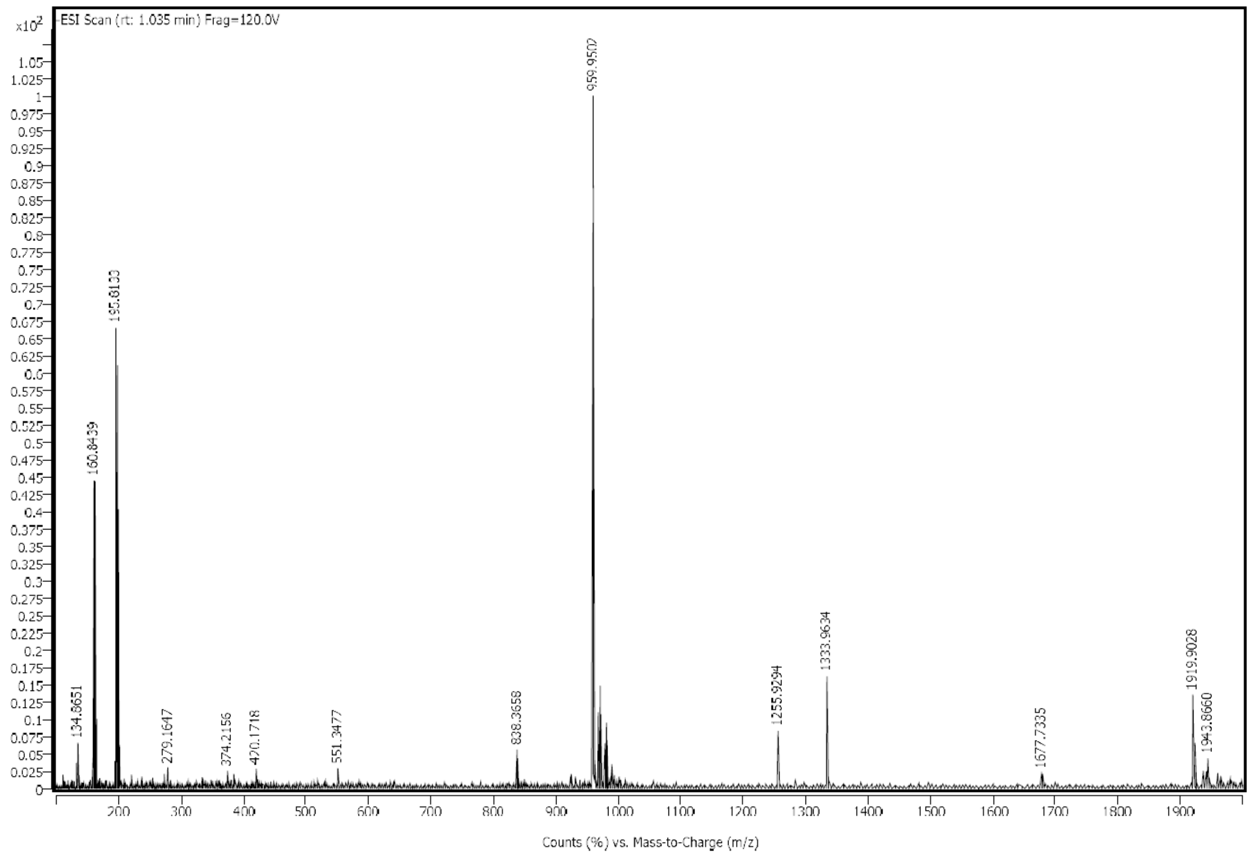

Fig. S8. Mass spectrum (HRMS ESI) of TOPA-paco.

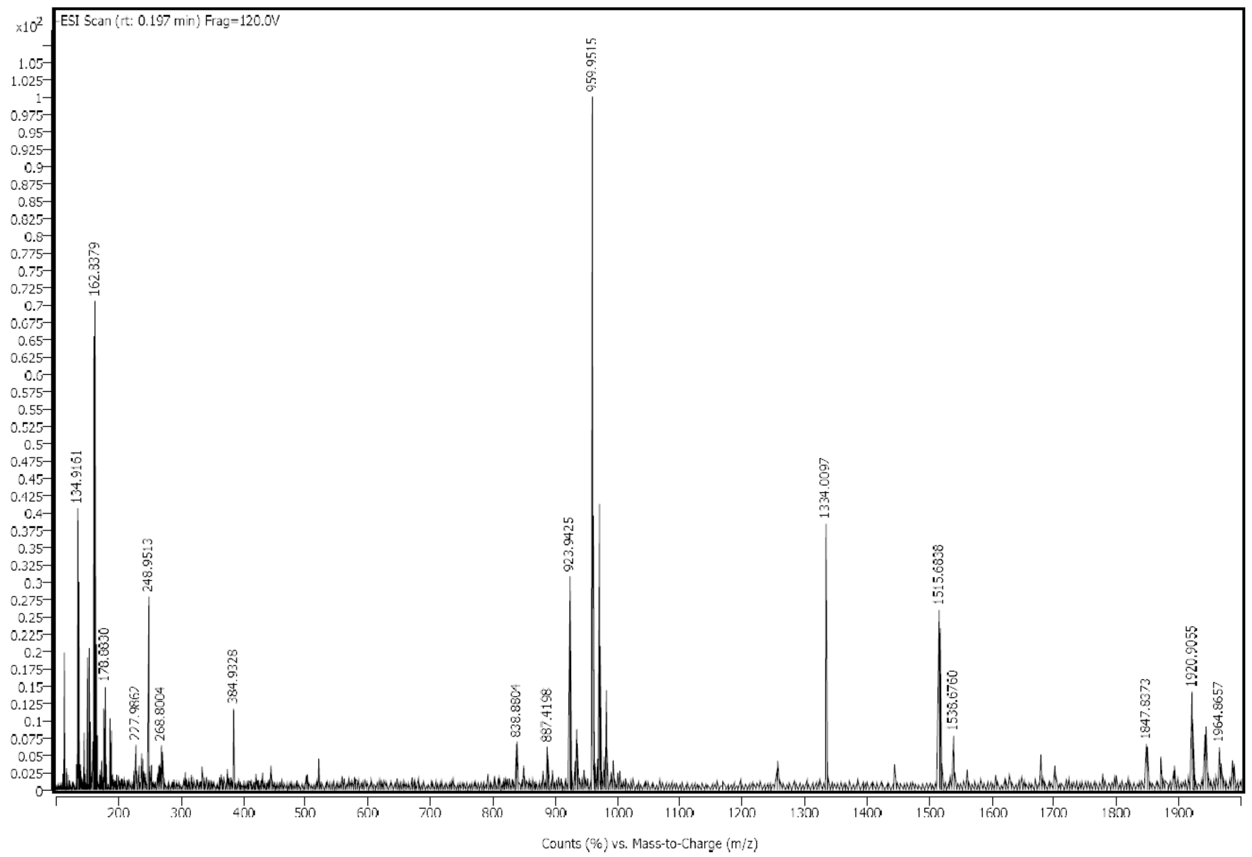

Fig. S9. Mass spectrum (HRMS ESI) of TOPA-alt.

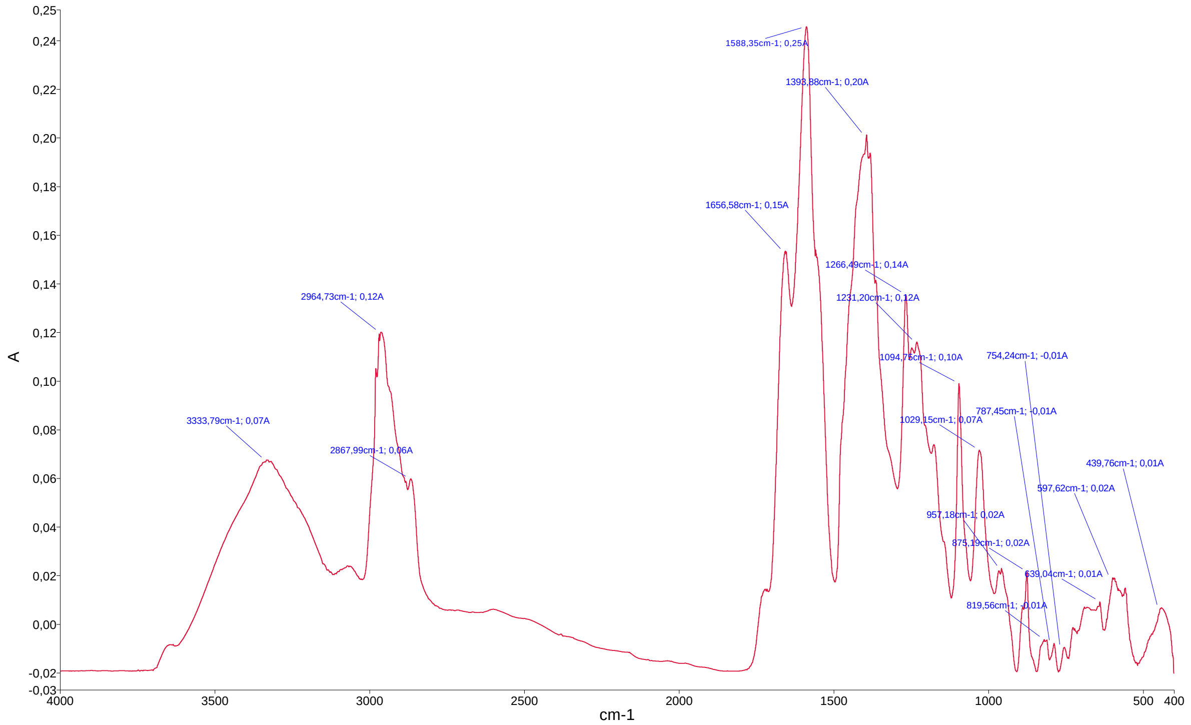

Fig. S10. FTIR spectrum of TOPA-cone.

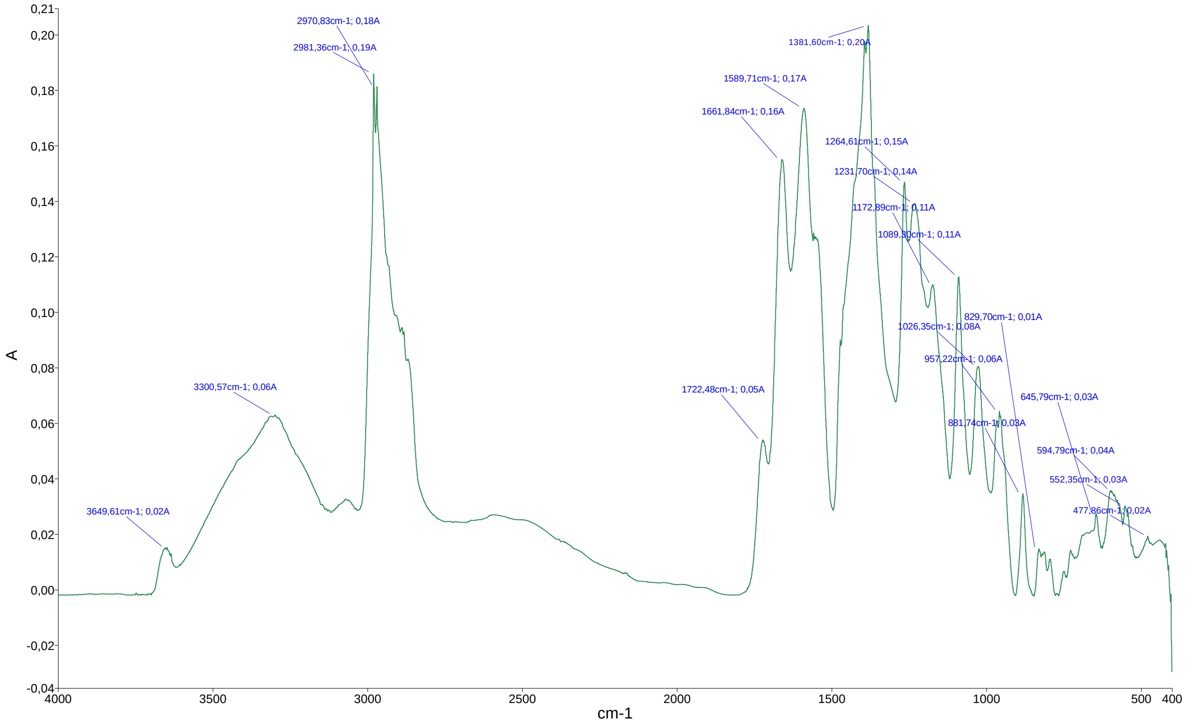

Fig. S11. FTIR spectrum of TOPA-paco.

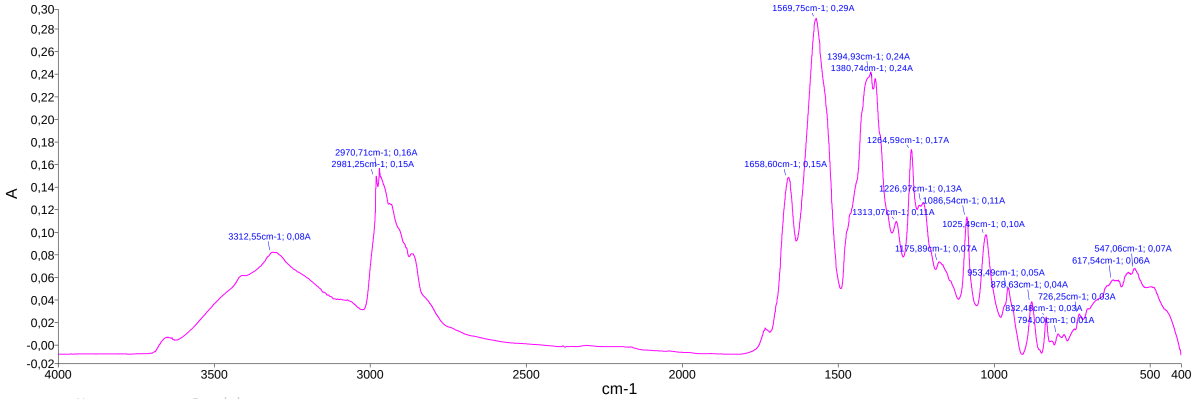

Fig. S12. FTIR spectrum of TOPA-alt.

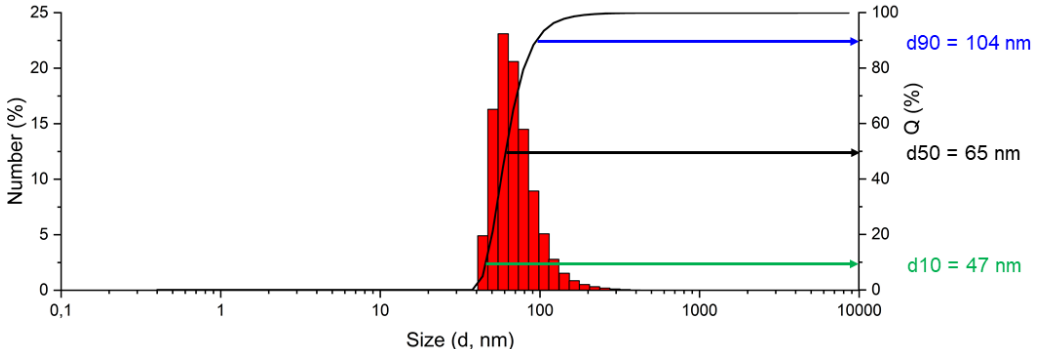

Fig. S13. Size distribution of particles by number for macrocycle TOPA-cone (1 µM) in H_2O.

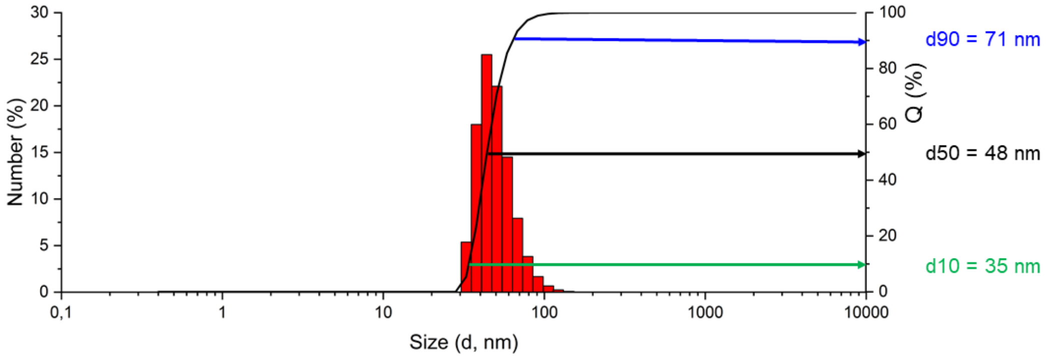

Fig. S14. Size distribution of particles by number for macrocycle TOPA-cone (10 µM) in H_2O.

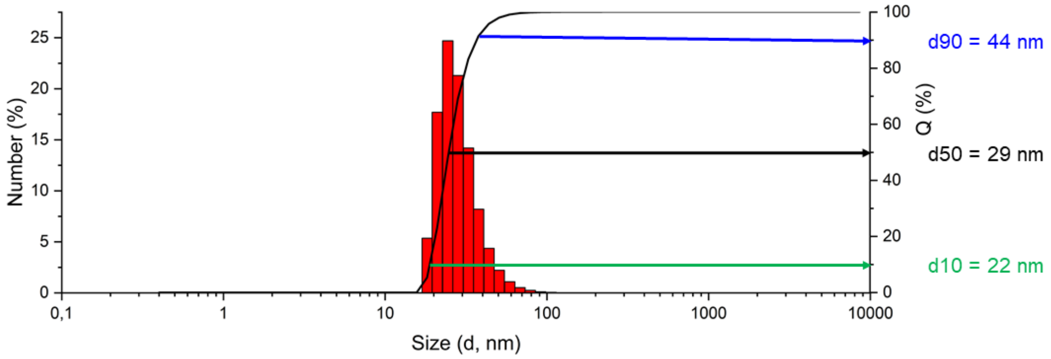

Fig. S15. Size distribution of particles by number for macrocycle TOPA-cone (100 µM) in H_2O.

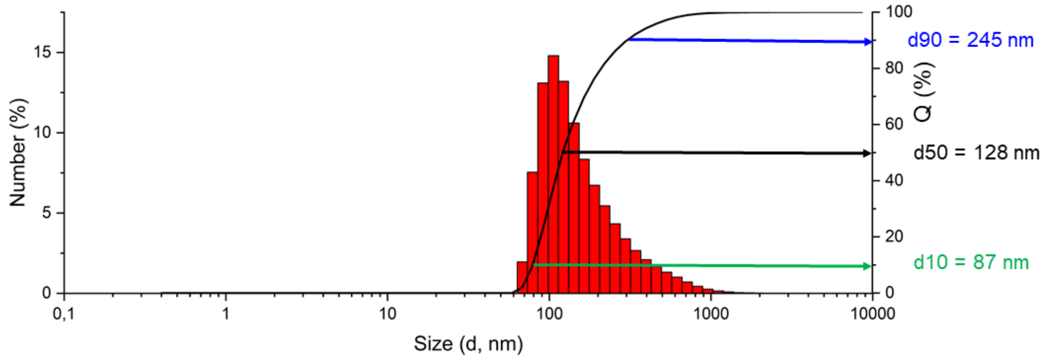

Fig. S16. Size distribution of particles by number for macrocycle TOPA-paco (1 µM) in H_2O.

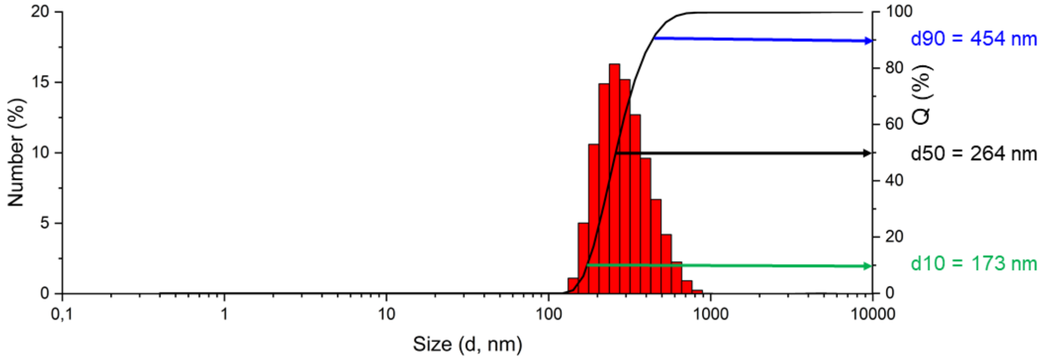

Fig. S17. Size distribution of particles by number for macrocycle TOPA-paco (10 µM) in H_2O.

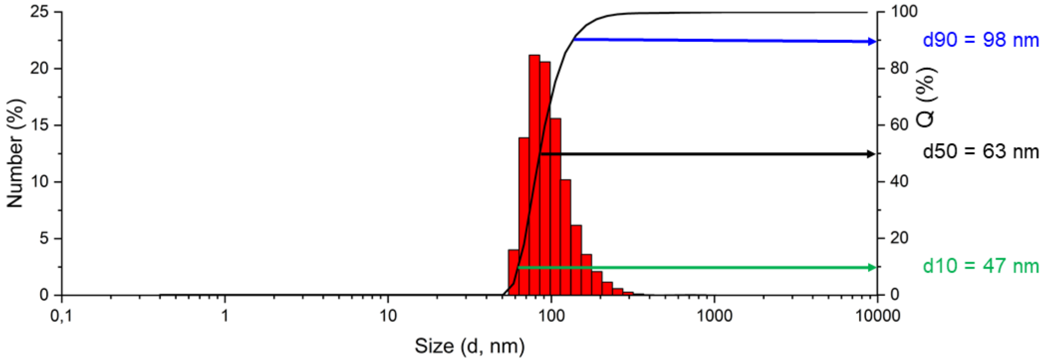

Fig. S18. Size distribution of particles by number for macrocycle TOPA-paco (100 µM) in H_2O.

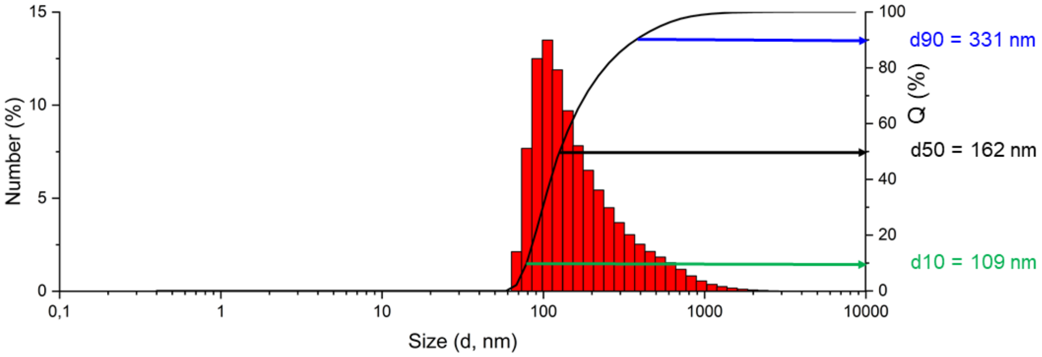

Fig. S19. Size distribution of particles by number for macrocycle TOPA-alt (1 μM) in H_2O.

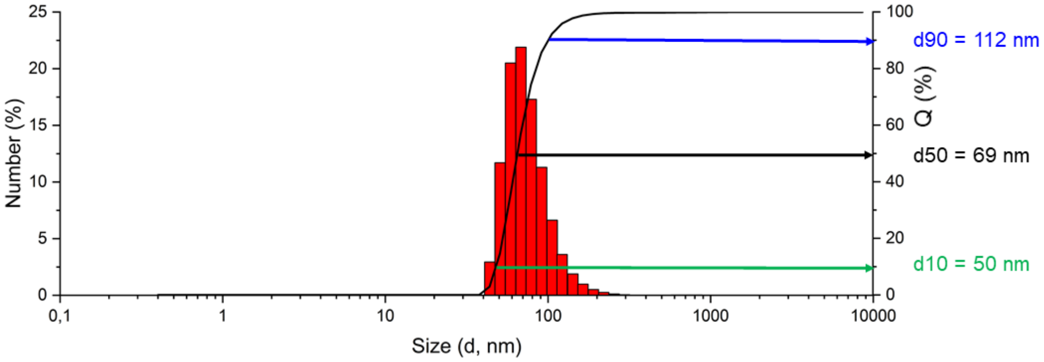

Fig. S20. Size distribution of particles by number for macrocycle TOPA-alt (10 μM) in H_2O.

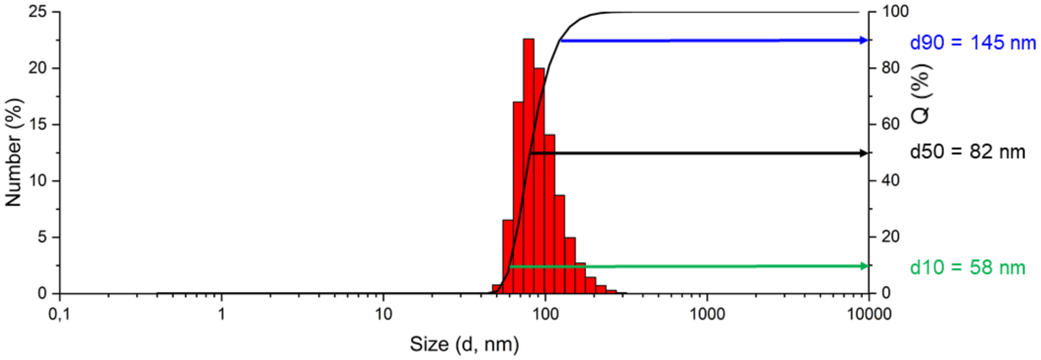

Fig. S21. Size distribution of particles by number for macrocycle TOPA-alt (100 μM) in H_2O.

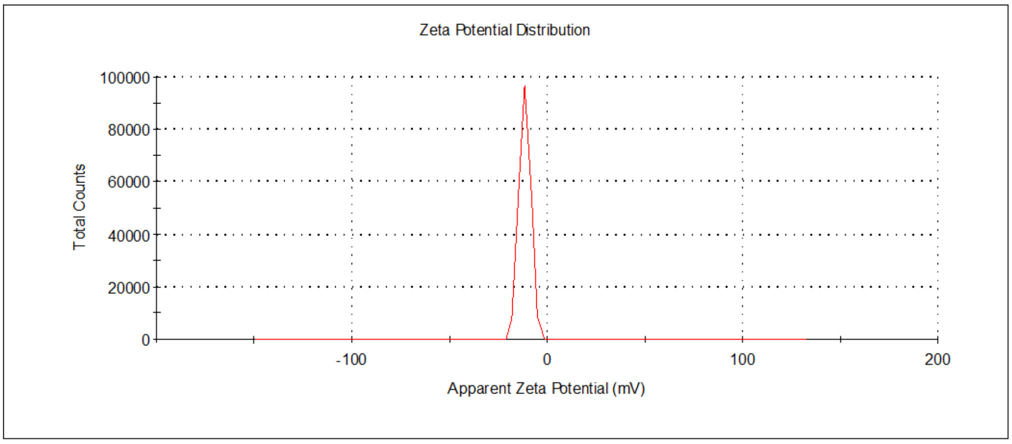

Fig. S22. Zeta potential distribution of the particles for macrocycle TOPA-cone (1 μM) in H_2O.

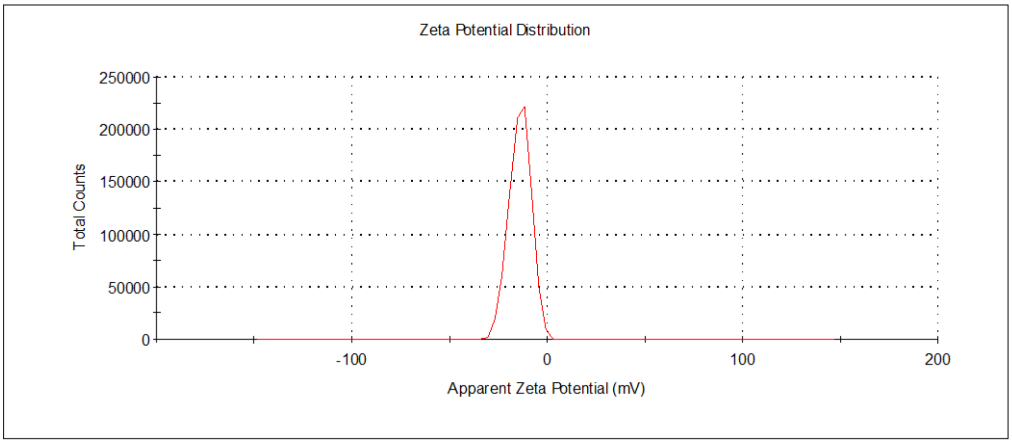

Fig. S23. Zeta potential distribution of the particles for macrocycle TOPA-cone (10 μM) in H_2O.

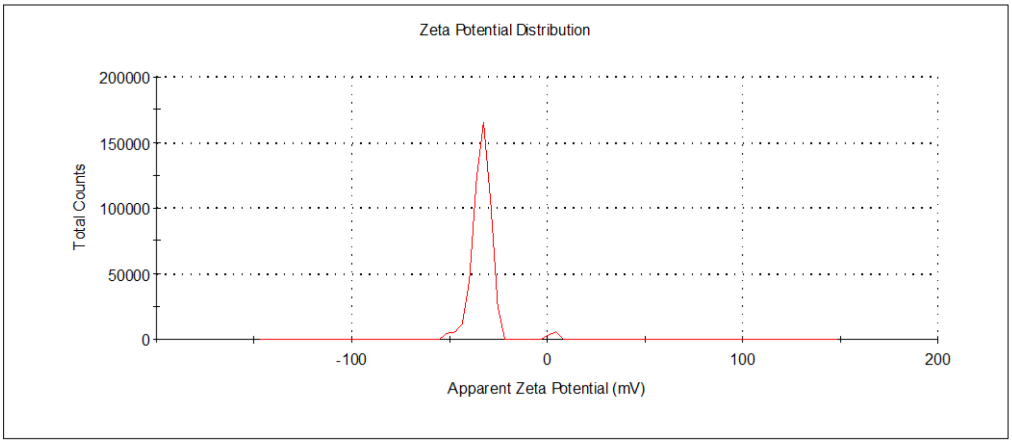

Fig. S24. Zeta potential distribution of the particles for macrocycle TOPA-cone (100 μM) in H_2O.

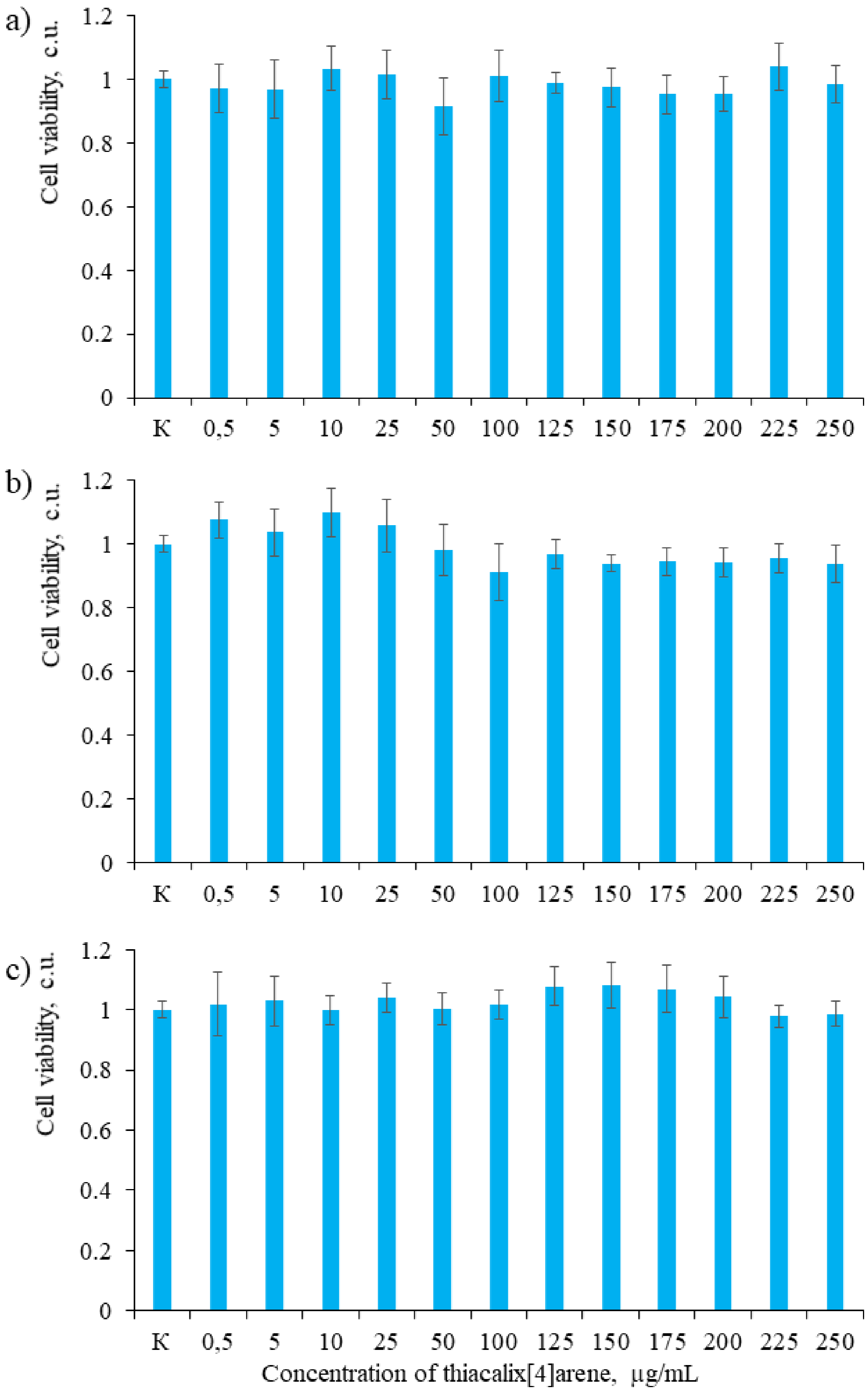

Fig. S25. Cytotoxicity of thiacalix[4]arenes on A549 cell line: a) TOPA-cone; b) TOPA-paco; c) TOPA-alt.

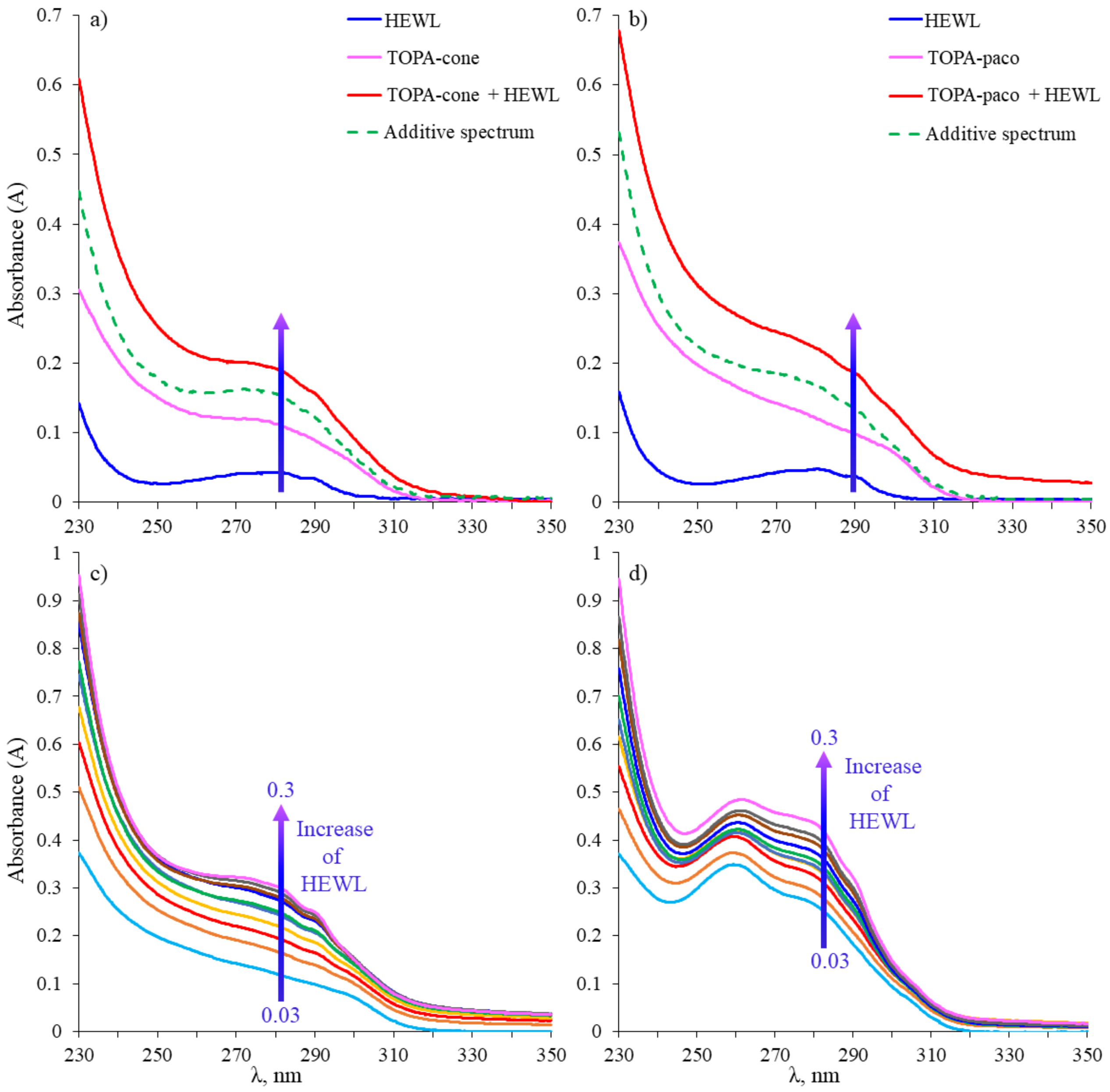

Fig. S26. Electron absorption spectra of: a) 10-fold excess of TOPA-cone with HEWL ($C_{TOPA\text{-}cone}$ = 10 μM, C_{HEWL} = 1 μM) in H_2O; b) 10-fold excess of TOPA-paco with HEWL ($C_{TOPA\text{-}paco}$ = 10 μM, C_{HEWL} = 1 μM) in H_2O; c) TOPA-paco with different concentrations of HEWL (from 0.03 to 0.3 excess) in H_2O; d) TOPA-alt with different concentrations of HEWL (from 0.03 to 0.3 excess) in H_2O.

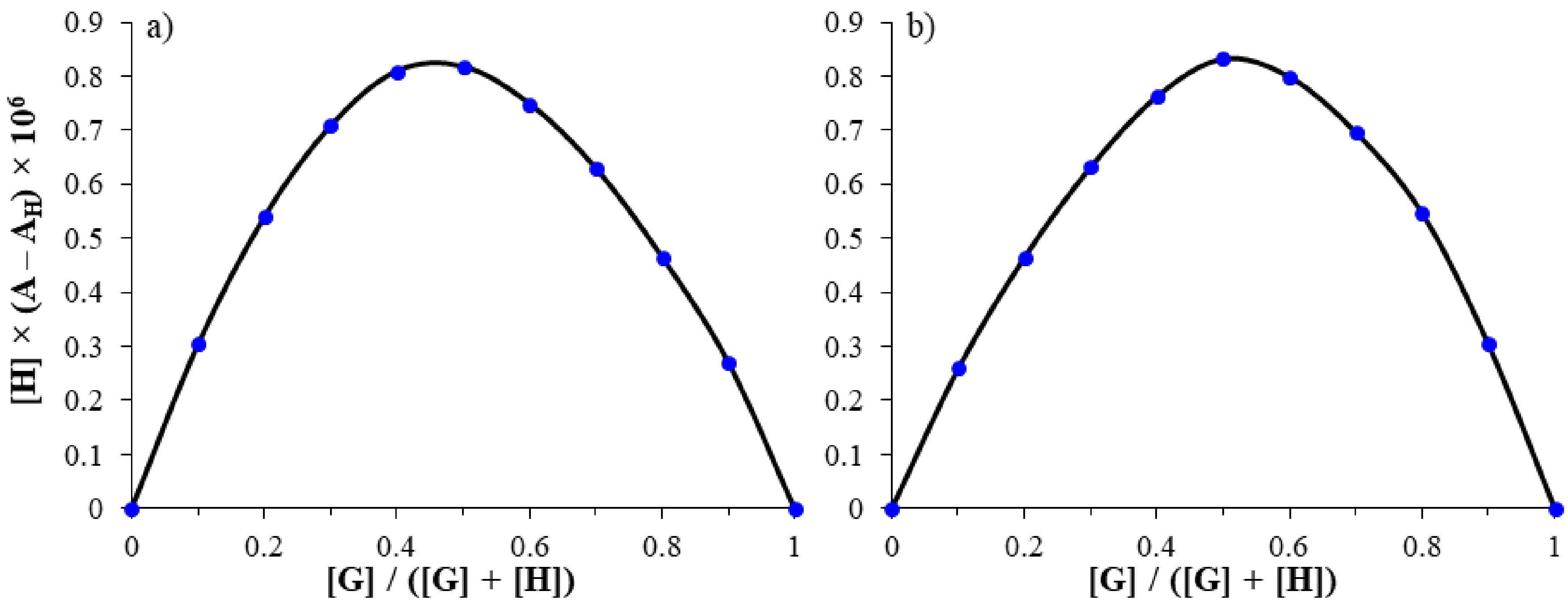

Fig. S27. Job's plot of: a) TOPA-paco and HEWL in H_2O (1:1); b) TOPA-alt and HEWL in H_2O (1:1).

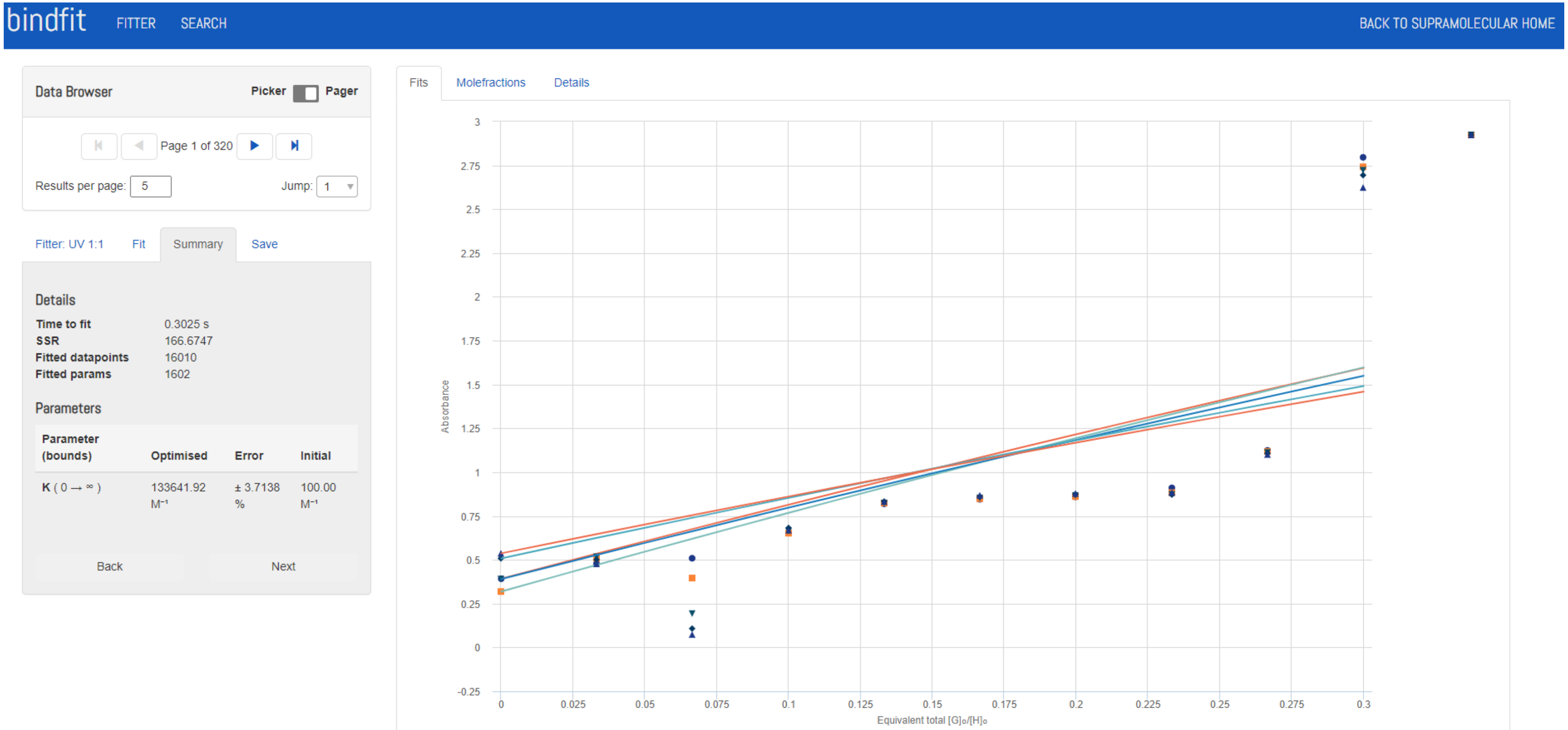

Fig. S28. Bindfit (Fit data to 1:1 Host-Guest equilibria). Screenshots taken from the summary window of the website supramolecular.org. This screenshots shows the raw data for florescence titration of TOPA-cone with HEWL in H_2O, the data fitted to 1:1 binding model.

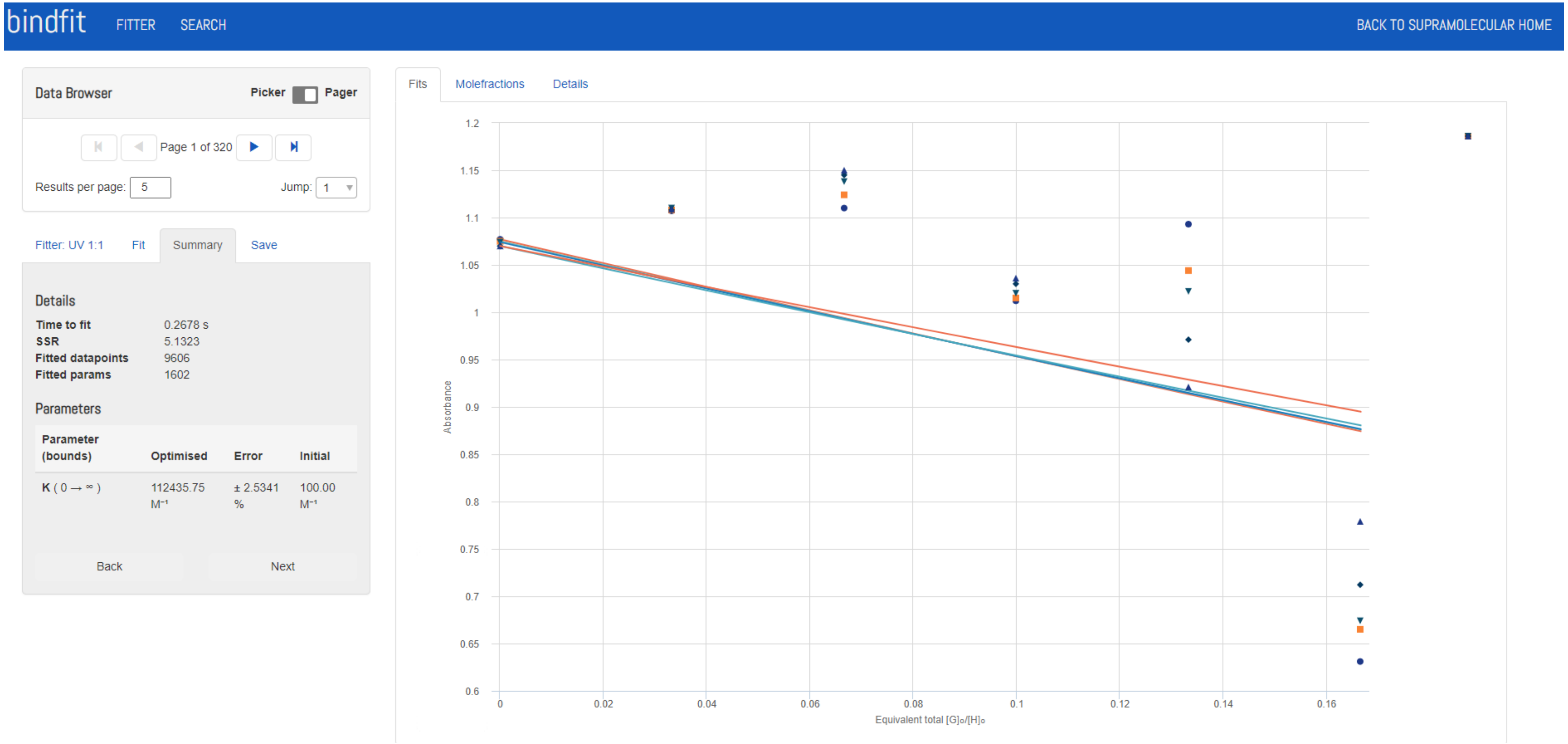

Fig. S29. Bindfit (Fit data to 1:1 Host-Guest equilibria). Screenshots taken from the summary window of the website supramolecular.org. This screenshots shows the raw data for florescence titration of TOPA-paco with HEWL in H_2O, the data fitted to 1:1 binding model.

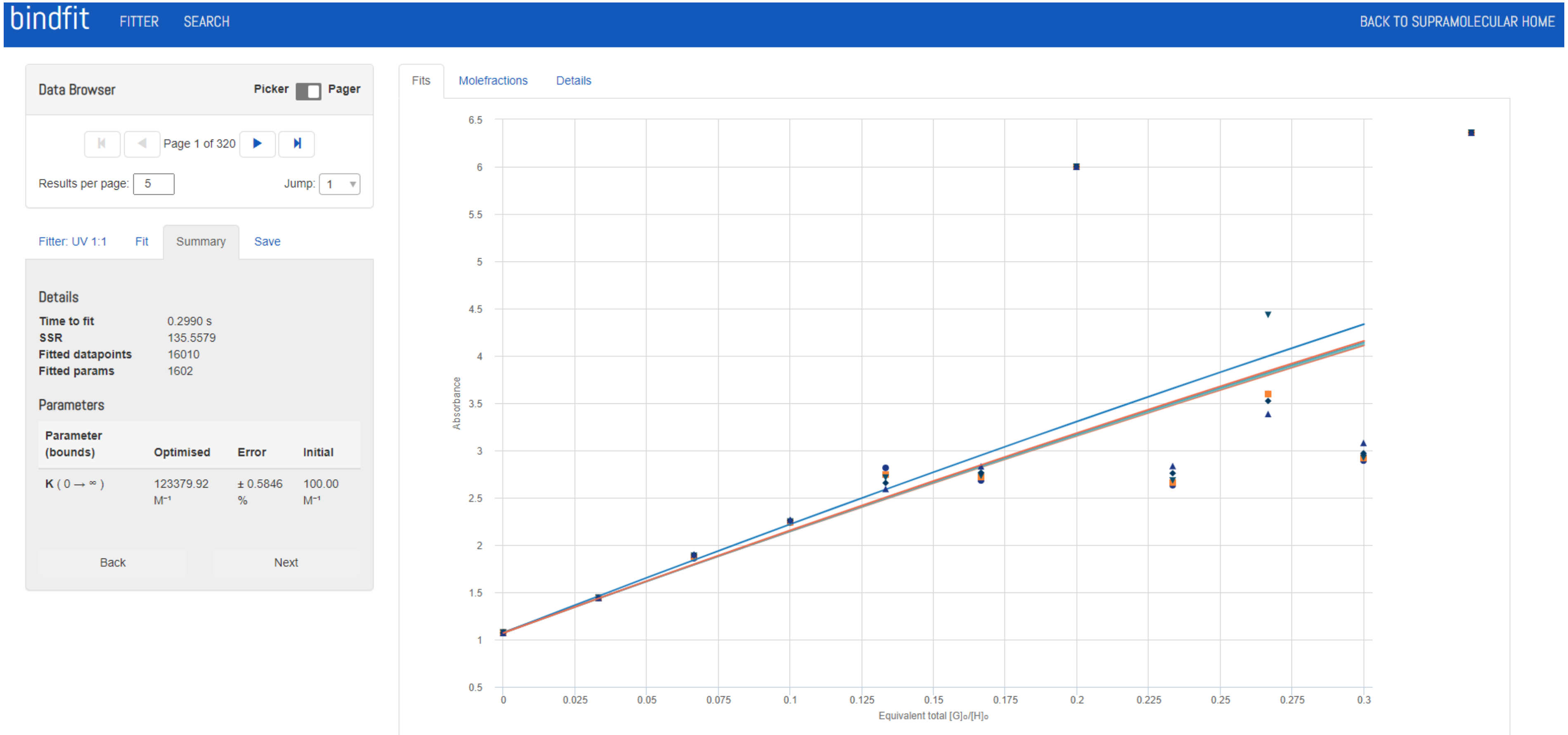

Fig. S30. Bindfit (Fit data to 1:1 Host-Guest equilibria). Screenshots taken from the summary window of the website supramolecular.org. This screenshots shows the raw data for florescence titration of TOPA-alt with HEWL in H_2O, the data fitted to 1:1 binding model.

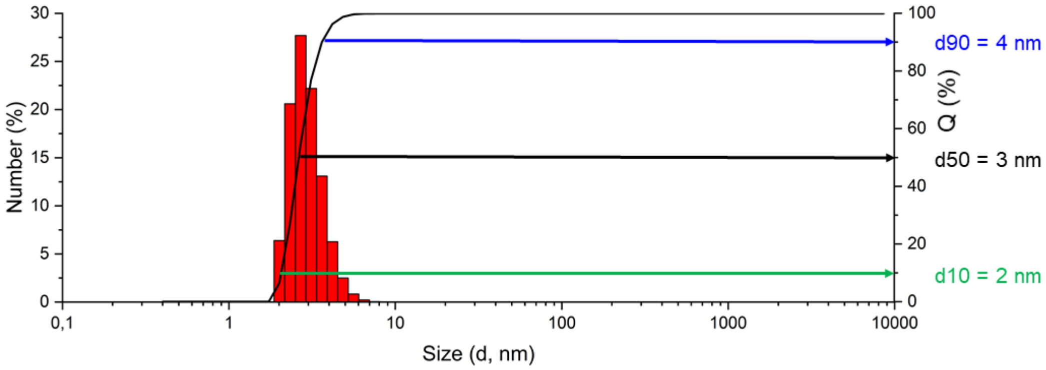

Fig. S31. Size distribution of particles by number for HEWL (C_{HEWL} = 10 µM) in H_2O.

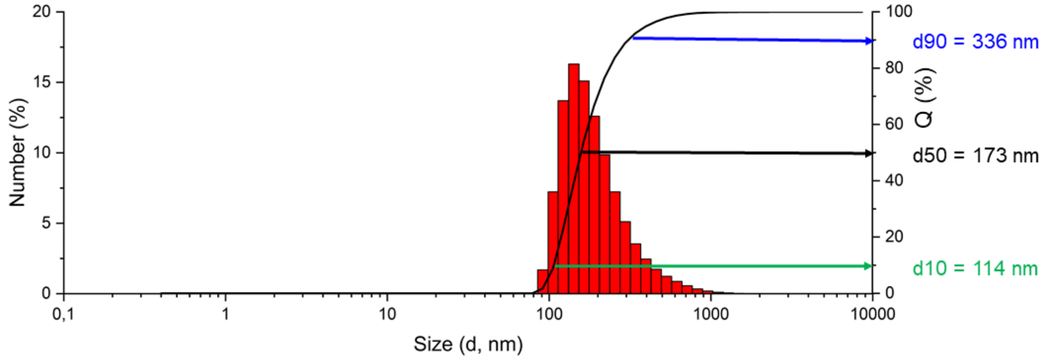

Fig. S32. Size distribution of particles by number for TOPA-cone / HEWL in equimolar ratio ($C_{TOPA\text{-}cone}$ = C_{HEWL} = 10 µM) in H_2O.

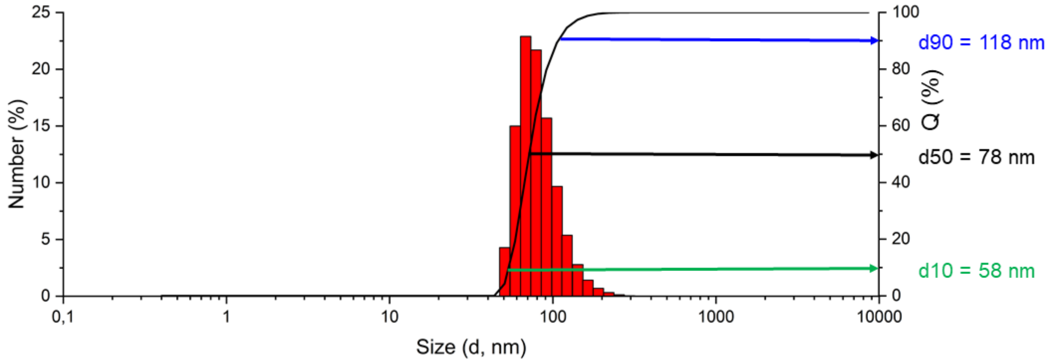

Fig. S33. Size distribution of particles by number for TOPA-paco / HEWL in equimolar ratio ($C_{TOPA\text{-}paco}$ = C_{HEWL} = 10 µM) in H_2O.

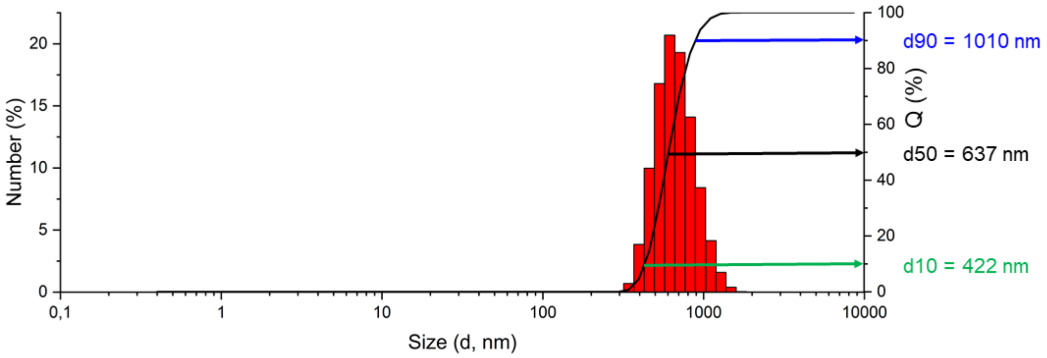

Fig. S34. Size distribution of particles by number for TOPA-alt / HEWL in equimolar ratio ($C_{TOPA\text{-}alt} = C_{HEWL} = 10$ µM) in H_2O.

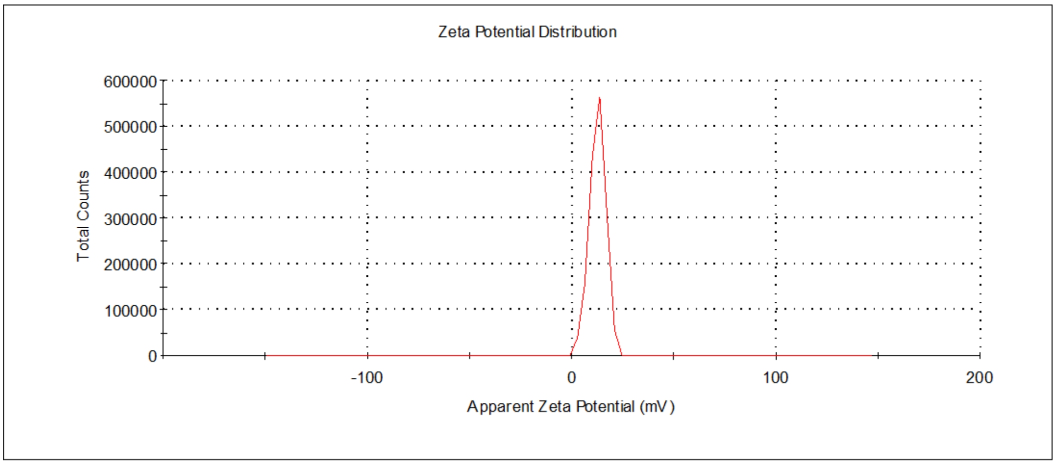

Fig. S35. Zeta potential distribution of the particles for TOPA-cone / HEWL in equimolar ratio ($C_{TOPA\text{-}cone} = C_{HEWL} = 10$ µM) in H_2O.

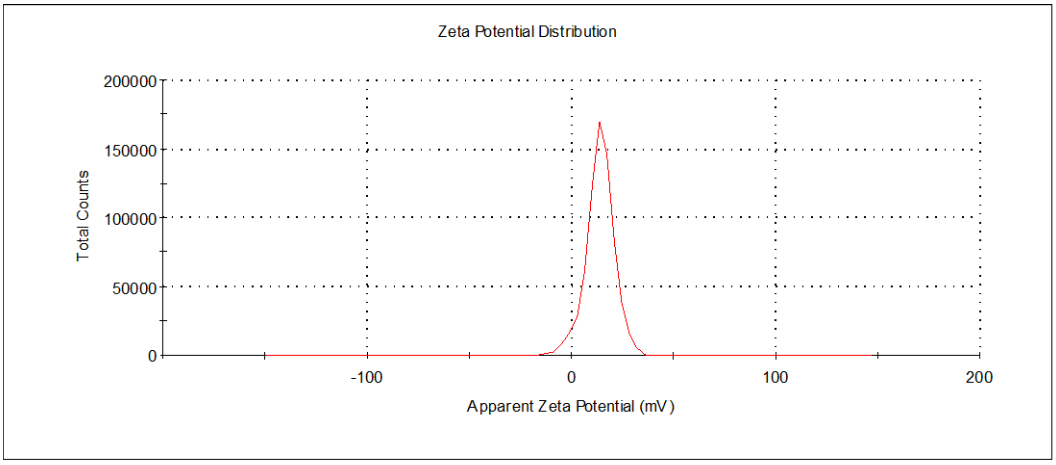

Fig. S36. Zeta potential distribution of the particles for TOPA-paco / HEWL in equimolar ratio ($C_{TOPA\text{-}paco} = C_{HEWL} = 10$ µM) in H_2O.

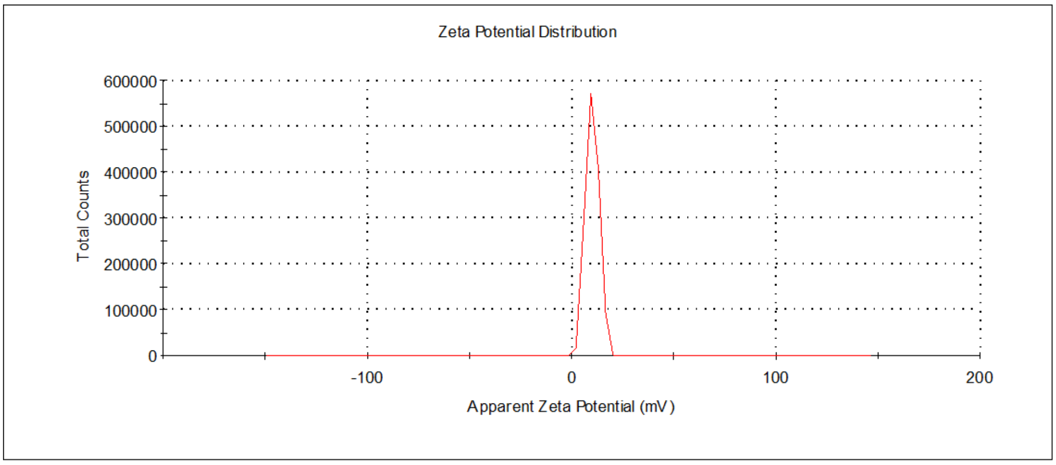

Fig. S37. Zeta potential distribution of the particles for TOPA-alt / HEWL in equimolar ratio ($C_{TOPA\text{-}alt}$ = C_{HEWL} = 10 μM) in H_2O.

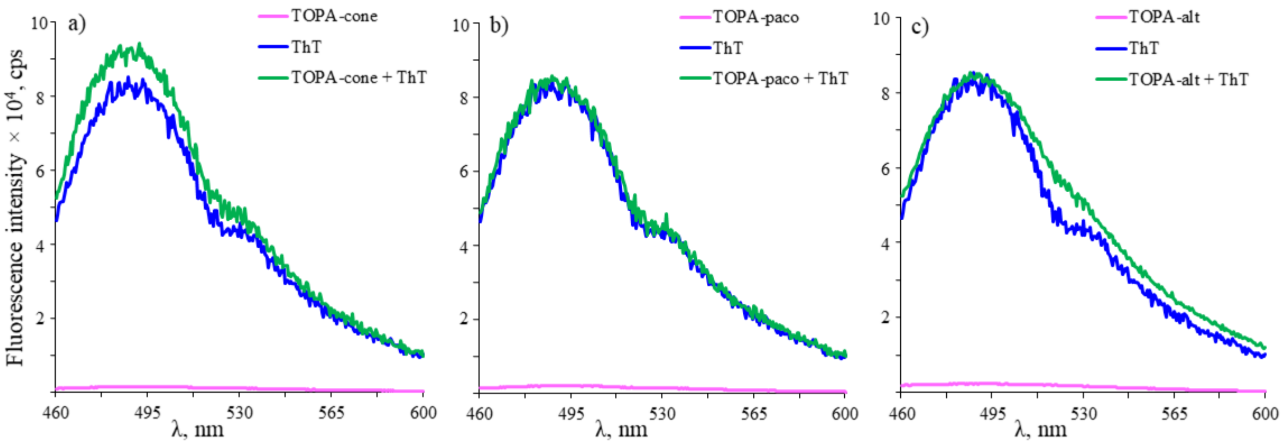

Fig. S38. Fluorescence spectra of: a) TOPA-cone with ThT; b) TOPA-paco with ThT; c) TOPA-alt with ThT in 20mM PBS ($C_{macrocycle}$ = C_{ThT} = 10 μM).

Peak Analysis

Data Set:[Book3]Sheet1!AG"Normalized Y5" Date:12.04.2024
BaseLine:Spline
Chi^2=1,19593E-03 Adj. R-Square=9,98596E-01 # of Data Points=1711
SS=2,02829E+00 Degrees of Freedom=1696

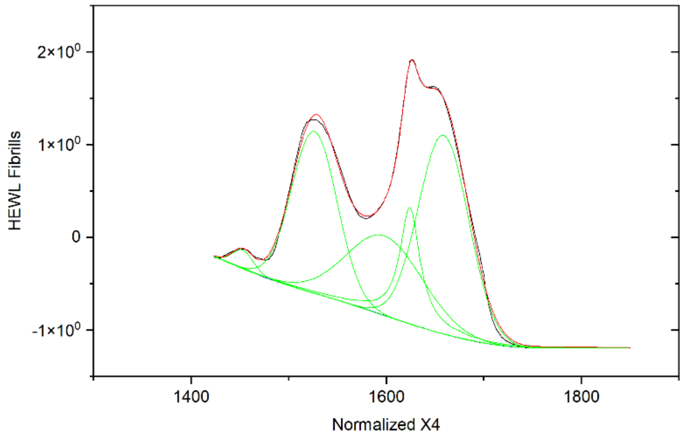

Fitting Results

Peak Index	Peak Type	Area Intg	FWHM	Max Height	Center Grvty	Area IntgP
1	Lorentz	5,36958	25,19841	0,20065	1452,82103	1,42184
2	Lorentz	102,21079	55,09781	1,74274	1526,1916	27,06486
3	Lorentz	84,23971	92,41946	0,85629	1598,04379	22,30622
4	Lorentz	47,24845	24,91947	1,25368	1624,08027	12,51113
5	Lorentz	138,58276	60,8429	2,13977	1658,63532	36,69596

Fig. S39. Fitting parameters in FTIR spectra of α-helix and β-sheet peak in HEWL fibrils. $IntArea\,\frac{\alpha-helices\ (peak\ 5)}{\beta-sheet\ (peak\ 4)} = 2.92$

Peak Analysis

Data Set:[Book3]Sheet1!AI"Normalized Y6" Date:12.04.2024
BaseLine:Spline
Chi^2=1,21774E-03 Adj. R-Square=9,98386E-01 # of Data Points=1711
SS=2,06407E+00 Degrees of Freedom=1695

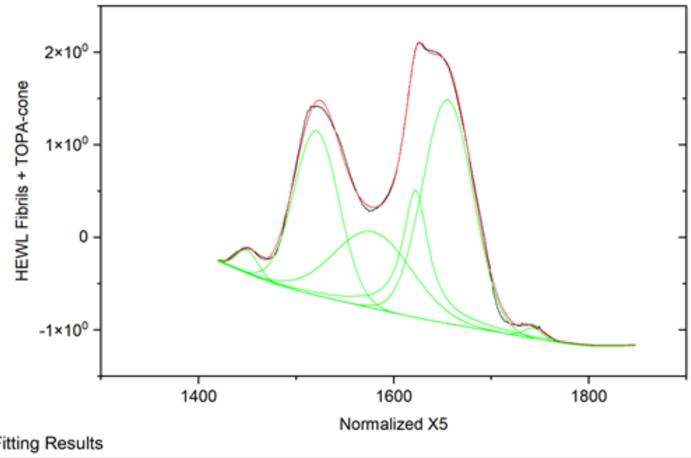

Fitting Results

Peak Index	Peak Type	Area Intg	FWHM	Max Height	Center Grvty	Area IntgP
1	Lorentz	5,86468	27,20958	0,20376	1451,39985	1,65628
2	Lorentz	85,66781	51,67518	1,55741	1524,1268	24,19404
3	Lorentz	49,01149	77,96856	0,59054	1563,81808	13,84167
4	Lorentz	73,00255	31,58546	1,54419	1622,81333	20,61715
5	Lorentz	140,53994	61,09247	2,16112	1656,35304	39,69085

Fig. S40. Fitting parameters in FTIR spectra of α-helix and β-sheet peak in HEWL fibrils with TOPA-cone. $IntArea\,\frac{\alpha-helices\ (peak\ 5)}{\beta-sheet\ (peak\ 4)} = 1.93$

Peak Analysis

Data Set:[Book3]Sheet1!AM"Normalized Y8" Date:12.04.2024
BaseLine:Spline
Chi^2=1,13066E-03 Adj. R-Square=9,98468E-01 # of Data Points=1711
SS=1,91420E+00 Degrees of Freedom=1693

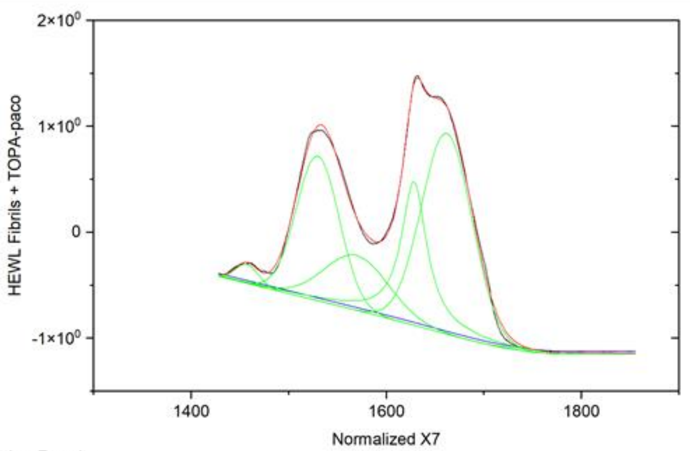

Fitting Results

Peak Index	Peak Type	Area Intg	FWHM	Max Height	Center Grvty	Area IntgP
1	Lorentz	6,00941	25,84521	0,21914	1452,45494	1,65372
2	Lorentz	89,70953	54,28964	1,55236	1523,46304	24,68701
3	Lorentz	72,20196	93,76053	0,72345	1581,24536	19,86913
4	Lorentz	56,67403	31,50808	1,20153	1624,90612	15,59603
5	Lorentz	136,38141	60,68255	2,11134	1657,87618	37,53057
6	Lorentz	2,41118	22,64737	0,10002	1745,78586	0,66353

Fig. S41. Fitting parameters in FTIR spectra of α-helix and β-sheet peak in HEWL fibrils with TOPA-paco. $IntArea\,\frac{\alpha\text{−}helices\ (peak\ 5)}{\beta\text{−}sheet\ (peak\ 4)} = 2.40$

Peak Analysis

Data Set:[Book3]Sheet1!AK"Normalized Y7" Date:12.04.2024
BaseLine:Spline
Chi^2=1,06520E-03 Adj. R-Square=9,98652E-01 # of Data Points=1711
SS=1,80658E+00 Degrees of Freedom=1696

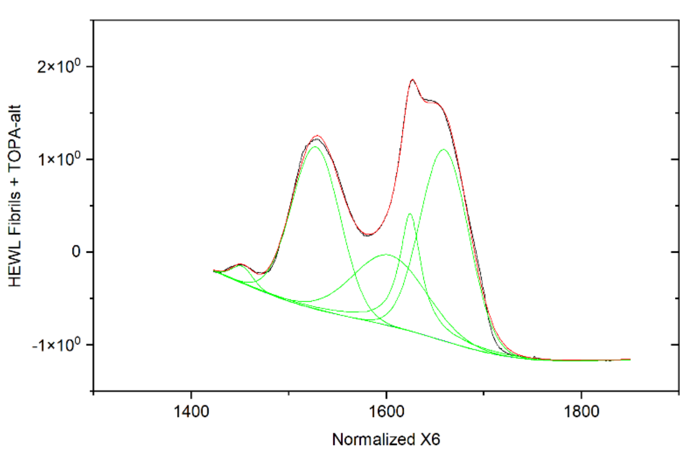

Fitting Results

Peak Index	Peak Type	Area Intg	FWHM	Max Height	Center Grvty	Area IntgP
1	Lorentz	4,86454	25,46501	0,17999	1452,29546	1,33853
2	Lorentz	108,83796	58,49499	1,74797	1527,78281	29,94796
3	Lorentz	71,3617	88,04415	0,76143	1603,57424	19,63595
4	Lorentz	50,55864	26,48537	1,26525	1624,50317	13,91176
5	Lorentz	127,80081	58,23398	2,0617	1659,49228	35,16579

Fig. S42. Fitting parameters in FTIR spectra of α-helix and β-sheet peak in HEWL fibrils with TOPA-alt. $IntArea\,\frac{\alpha\text{−}helices\ (peak\ 5)}{\beta\text{−}sheet\ (peak\ 4)} = 2.57$

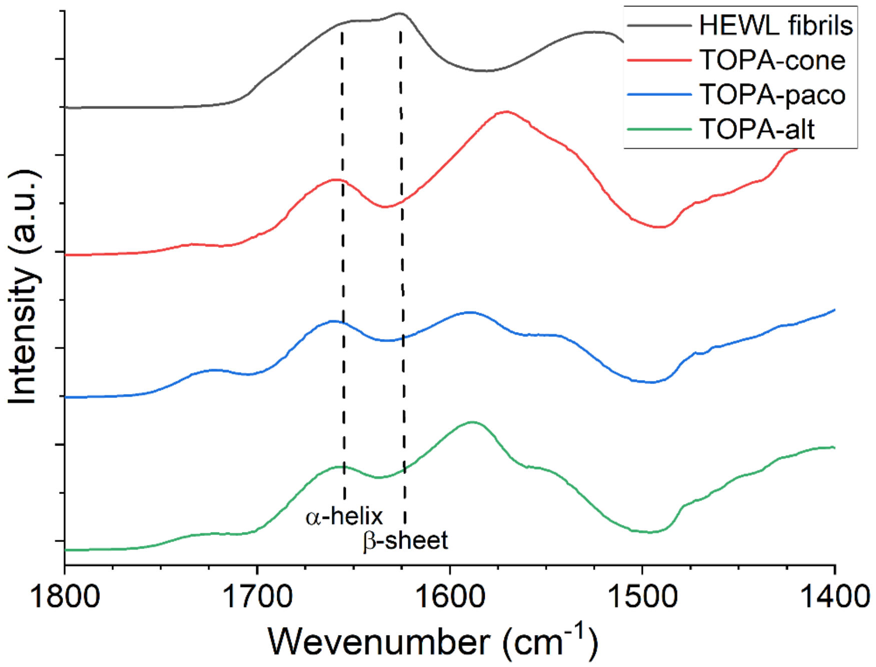

Fig. S43. FTIR spectra of thin films of HEWL fibrils and TOPA macrocycles.

References

[S1] T. Mosmann, Rapid colorimetric assay for cellular growth and survival: Application to proliferation and cytotoxicity assays, J. Immunol. Methods. 65 (1983) 55–63. https://doi.org/10.1016/0022-1759(83)90303-4

[S2] C.N. Pace, F. Vajdos, L. Fee, G. Grimsley, T. Gray, How to measure and predict the molar absorption coefficient of a protein, Protein Sci. 4 (1995) 2411-2423. https://doi.org/10.1002/pro.5560041120
